# Chemical Composition, Antioxidant and Antimicrobial Activity of *Pericarpium Citri Reticulatae* Essential Oil

**DOI:** 10.3390/molecules16054082

**Published:** 2011-05-18

**Authors:** Bei Gao, Yulong Chen, Mingwei Zhang, Yujuan Xu, Siyi Pan

**Affiliations:** 1 College of Food Science and Technology, Huazhong Agricultural University, Wuhan 430070, China; 2 Sericulture & Agro-Food Processing Research Institute, Guangdong Academy of Agricultural Sciences, Guangzhou 510610, China

**Keywords:** *Pericarpium Citri Reticulatae*, Chachi, chemical composition, antioxidant activity, antimicrobial activity

## Abstract

The chemical composition, antioxidant and antimicrobial activity of *Pericarpium Citri Reticulatae* (*PCR*) essential oil obtained using an improved Clevenger type apparatus were studied. Among the five different *PCR*s examined the highest yield of essential oil was found in Chachi 2004 (harvested and stored in 2004) and the lowest in Chachi 2008 (harvested and stored in 2008). Fifty three different volatile compounds were determined, including terpenic hydrocarbons, alcohols, aldehydes, ketones and esters. D-limonene, one of terpenes, was the major constituent in *PCR*. The antioxidant capacity of *PCR* essential oil varied considerably with the duration of storage time, and the oil from Chachi 1994 has the strongest ferric-reducing antioxidant power. In addition, the essential oil possessed varying degrees of antimicrobial activity against Gram-positive bacteria (*Staphylococcus aureus*, *Bacillus subtilis*, *Bacillus cereus*), except *Streptococcus faecalis*, while had no effect on Gram-negative bacteria (*Escherichia coli*, *Enterobacter cloacae*).

## 1. Introduction

*Pericarpium Citri Reticulatae* (*PCR*), the dried ripe peel of mandarin (*Citrus reticulata* Blanco) and its cultivars, is acknowledged in the People’s Republic of China Pharmacopoeia, and has been used in traditional Chinese herbal medicine for a long time [1,2]. *PCR* “Chachi”, as the most popular type, has been used in foods and drugs due to its effectiveness as an antioxidant, in regulating qi (energy), normalizing the function of spleen and stomach, resolving phlegm, and so on [3,4,5]. The essential oil of *PCR* “Chachi”, as one of major bioactive compositions, perhaps accounts for the use of *PCR* as a Chinese herbal medicine.

At present, some studies on the fresh peel oils of different mandarins have been reported. The major monoterpenes of Turkish mandarin peel oil were found to be D-limonene (90.7%), γ-terpinene (3.9%), myrcene (2.1%), α-pinene (0.5%), sabinene (0.3%) [6]. The result is agreement with other literature. In Ponkan peel oil, where the monoterpene fraction accounts for more than 89.6% of the components, D-limonene was the most abundant component (80.3%), followed by γ-terpinene (4.7%), myrcene (2.1%) and α-pinene (1.2%) [7]. The major sesquiterpene component was (*E*)-β-farnesene (0.1%). The major oxygenated components found in the oil were octanal (0.2%), decanal (0.1%), linalool (0.4%), α-terpineol (0.1%), geranyl acetate (0.2%) and neryl acetate (0.1%) [6].

In addition, the essential oil of *PCR* has also been studied. The literature shows that the major components of *PCR* essential oil are D-limonene (75.28%), 1-methyl-4-(1-methylethyl)-1,4-cyclo- hexadiene (7.7%), β-myrcene (2.98%), α,α-4-trimethyl-3-cyclohexene-1-methanol (0.94%), 2-methoxy-4-vinylphenol (0.87%) and α-pinene (0.53%) [8]. Among the components, terpenes as the major components of the essential oil have effective antifungal and antioxidant activity [9,10,11]. Thus, the essential oil can partially replace chemical and synthetic agents with antioxidant and antimicrobial activity to avoid the toxicities and carcinogenic effects of chemical and synthetic agents [12,13,14]. However, there are few reports on the variation of essential oil of *PCR* after different storage times in relation to the function of *PCRs*. In traditional Chinese medicine, the quality of *PCR* “Chachi” is regarded to increase with the storage time, however, the reason for this remains unknown. Therefore, we focused our study on the chemical compositions, antioxidant and antimicrobial activity of the essential oil in *PCR* “Chachi” to try to find the truth.

## 2. Results and Discussion

### 2.1. Yields Rates of the Essential Oil

At present, many researchers used Clevenger apparatus to extract essential oil from samples [15,16,17]. The yield of essential oil by an improved microwave Clevenger apparatus in 30 min was equivalent to that obtained after 3 h with a regular Clevenger apparatus, as reported by Ferhat *et al.* [18]. The high temperature processing of hydro-distilled extraction methods, such as Clevenger apparatus can result in degradation of thermally labile compounds [19]. In our work the essential oil of *PCR*s was extracted with a modified Clevenger-type apparatus with a water-cooled oil receiver to reduce formation of artifacts due to overheating during hydro-distillation; the yield rates were listed in Table 1. The results showed that the highest essential oil yield was found in Chachi 2004 and the lowest in Chachi 2008. The essential oil yield variation first went up and then down with the duration of storage time. Since all the five types of *PCRs* were harvested from the same cultivar in the same plantation, it was inferred that the variation on yield rate of essential oil could not be caused by the difference of harvested material for *PCRs*. Therefore, the increase of the essential oil from Chachi 2008 to Chachi 2004 may be attributed to the transformation of some metabolites to the essential oil by way of secondary metabolism. The decrease in yield rate of essential oil with storage time from Chachi 2004 to Chachi 1994 probably happened because of high consumption of essential oil due to evaporation with the duration of storage time.

### 2.2. Linearity and Recovery of Standards

A standard mixture of seven compounds found in *PCR* oil was used to test linearity and recovery. Five levels of each analyte were prepared for plotting standard calibration curves. The concentration ranges, regression equations, R^2^ values, recoveries are shown in Table 2. The results showed a good linear behavior in the concentration ranges. Furfural showed the best linearity (R^2^ = 0.9983) and the least linearity was obtained for *α*-pinene (R^2^ = 0.9588). Recoveries were performed to test the accuracy of the method. As shown in Table 2, the average recoveries of standard compounds ranged from 84.9% to 113.7%. The results demonstrated that the method was applicable for the analysis of *PCR* oil because of good linearity and recoveries. It was agreement with the report by Mirhosseini *et al*. [20], while the report by Ibáñez *et al*. showed that the recoveries of 3-methylbutanol and ethyl-hexanoate ranged from 10 to 45%, and the recovery of hydrocarbons ranged from 4 to 25% [21].

### 2.3. Sample Analysis

**Figure 1:**
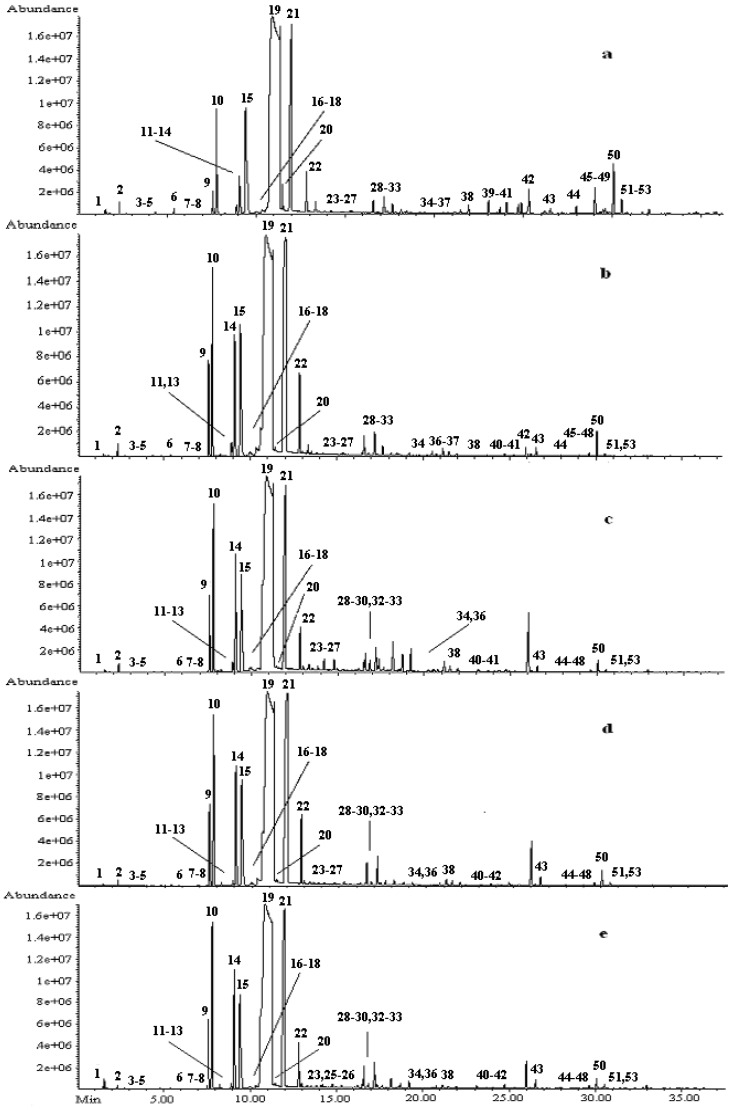
GC chromatogram of *PCR* essential oil at different storage time **a**: Chachi 2008, **b**: Chachi 2004, **c**: Chachi 2001, **d**: Chachi 1998, **e**: Chachi 1994. Component numbers in the chromatogram come from Table 1.

*PCR* oil with five different storage times was analyzed to determine the composition of volatile compounds (Table 1 and Figure 1). Fifty three volatile compounds from the *PCR* oil were detected by GC-MS. The volatiles detected included terpenic hydrocarbons, alcohols, aldehydes, ketones and esters. A total of 61 and 59 compounds in the essential oil of *Pericarpium Citri Reticulatae Viride* (*PCRV*) and *PCR* determined were previously reported by Wang *et al*. [22]. There were 53, 48, 46, 47 and 45 volatile compounds identified in Chachi 2008, Chachi 2004, Chachi 2001, Chachi 1998 and Chachi 1994 respectively. Among these volatile compounds, seven compounds were identified by using chemical standards and the others were identified tentatively. The terpenes in volatile compounds were major components, and the composition and concentration of the terpenes were observed to differ with storage time (Table 1). Among the samples of five different storage times, the terpenes content of Chachi 2008 was highest. D-limonene was the predominant compound in the *PCR*s. It was in agreement with olfaction tests. D-limonene was also identified as the major component, accounting for 65.61-83.14% in *PCRV*s and *PCR*s reported by Wang *et al*. [22]. In addition, the concentrations of several volatile compounds such as β-myrcene, γ-terpinene, terpinolene and β-pinene in *PCR*s were detected to be relatively high.

### 2.4. Antioxidant Activity

Antioxidation is a complex process usually occurring through several mechanisms. The evaluation of the antioxidant activity for pure compounds or extracts should be carried out by more than one test method [39]. For the scavenging of DPPH radicals, the IC_50_ values (defined as the concentration of sample at which the inhibition percentage reached 50%) of essential oil were different (Table 3). The IC_50_ value of Vc was 3.14 ± 0.21 μg/mL. DPPH scavenging activity of those samples followed the order: Chachi 2008 > Chachi 2001 > Chachi 1998 > Chachi 2004 > Chachi 1994. The scavenging activity of Chachi 2008 was significantly (P < 0.05) stronger than that of the others. For the reducing power, Table 3 shows great differences in total antioxidant activity measured by the FRAP method. Chachi 1994 had the highest value of 26.89 ± 1.11 μmol TE/g, while Chachi 2008 had the lowest value of 11.36 ± 1.50 μmol TE/g. The rank order was Chachi 1994 > Chachi 1998 > Chachi 2001 > Chachi 2004 > Chachi 2008. The results showed that the reducing power of *PCR* essential oil from different storage times increased with the duration of *PCR* storage time. It is probably related to the increment of some components such as α-pinene and β-pinene during storage time. For the scavenging ABTS·^+^ radicals, the results showed that the inhibitory potentials followed the order: Chachi 2001 > Chachi 1998 > Chachi 2004 > Chachi 2008 > Chachi 1994. Thus the scavenging ABTS·^+^ radicals of *PCR* essential oil differed with the storage time. Although there were few literatures about the antioxidant activity of *PCR* essential oil, the antioxidant activity of *PCR* extract has been reported. According to Su *et al.* [40], the reducing power of *PCR* extracts increased with concentration (0-0.3 mg/mL) and the EC_50_ is 0.25 ± 0.02. However, the correlation between EC_50_ values of reducing power and total phenolic contents of *PCR* tested was low and not significant. Thus the reducing power of *PCR* extract might not attribute to the main phenolic components. Meanwhile, *PCR* extract had lower hydrogen peroxide-scavenging effect.

In general, the antioxidant capacities of *PCR* essential oil varied considerably with the storage time while Chachi 1994 has best antioxidant by FRAP. It may partially demonstrate *PCR* as a genuine medicinal herb why the pharmacologic activity is better with the duration of storage time.

### 2.5. Antimicrobial Activity

In Table 4, the *PCR* essential oil of different storage times were tested for antimicrobial activity by using 11 strains of microbes, viz. *Salmonella lignieres* (*S. lignieres*), *Escherichia coli* (*E. coli*), *Staphylococcus aureus* (*S. aureus*), *Bacillus subtilis* (*B. subtilis*), *Pseudomonas aeruginosa* (*P. aeruginosa*), *Bacillus cereus* (*B. cereus*), *Enterobacter cloacae* (*E. cloacae*), *Streptococcus faecalis* (*S. faecalis*), *Aspergillus flavus* (*A. flavus*), *Aspergillus niger* (*A. niger*), *Debaryomyces hansenii* (*D. hansenii*). The results showed that the essential oil possessed effective antimicrobial activity against Gram-positive bacteria (*S. aureus*, *B. subtilis*, *B. cereus*) in varying degrees except *S. faecalis*. The essential oil had no effect on Gram-negative bacteria (*E. coli*, *E. cloacae*). Those results were confirmed by literatures. It has been reported that the essential oil from citrus peel shows inhibition against all Gram-positive bacteria, yeast and mold [41,42,43]. *B. subtili*, *B. cereus*, *A. flavus* and *D. hansenii* were the most sensitive microorganisms to the *PCR* essential oil (diameter of inhibition zone ranging from 8.1 to 16.9 mm; MIC values ranging from 0.03 to 0.06 mg/mL). Gram-positive bacteria are more sensitive to essential oil than Gram-negative bacteria due to their outer membrane barriers [44]. Gram-positive bacteria are more susceptible since have only an outer peptidoglycan layer which is not an effective permeability barrier while Gram-negative bacteria have outer phospholipids membranes [45]. Some reports showed terpenes, which constitute the major part of citrus peel oil, have a strong antifungal and antioxidant activities [9,46]. α-Pinene (monoterpene hydrocarbon) had slight activity against a panel of microorganisms [47]. Despite slight activity, pinene-type monoterpenes could be responsible for the total activity spectrum. At present, the mechanism of terpenes action is not fully understood but is speculated to involve membrane disruption by the lipophilic compounds [48].

## 3. Experimental

### 3.1. Materials and Chemicals

Five types of *PCR*s (named as “Chachi + harvested time”) were purchased from “Xin Baotang Chen Pi” Co. Ltd., which was one of the biggest traders of *PCR* in Xinhui County, China. All five types of *PCR*s were harvested from the same “Chachi” cultivar (*Citrus reticulata* Blanco) in the same plantation. These samples were authenticated by Professor Jiang Yueming at South China Botanical Garden, Chinese Academy of Sciences. A voucher specimen of each type is deposited at the Sericulture & Agro-Food Processing Research Institute, Guangdong Academy of Agricultural Sciences. These samples were dried in the oven at 50 °C to constant weight and ground and stored in refrigerator at 4 °C before use.

1,1-Diphenyl-2-picrylhydrazyl (DPPH) was purchased from Wako Co. Ltd. 2,4,6-Tri-pyridyl-*s*-triazine (TPTZ) was purchased from Tokyo Kasei Kogyo Co. Ltd. ABTS was purchased from Amresco Co. Ltd. 6-Hydroxy-2,5,7,8-tetramethyl-2-chromanecarboxylic acid (Trolox), cyclohexanone and a mixture of aliphatic hydrocarbons (C_6_-C_22_) were obtained from Sigma Chemical Co. (Shanghai, China). The standard chemicals used for identification, such as D-limonene was obtained from Aladdin Chemistry Co. Ltd. (Shanghai, China). The aroma standards furfural, α-pinene, β-myrcene, terpinolene, decanal and β-caryophyllene were provided without charge from Gld-boton Essential Company (Shenzhen, China). All other chemicals, analytical grade, i.e. anhydrous sodium carbonate, trichloroacetic acid (TCA), potassium dihydrogen phosphate, potassium hydrogen phosphate, potassium persulfate, dimethyl sulfoxide (DMSO), and methanol used in this study were purchased from Guangzhou Chemical Reagant Plant (Guangzhou, China). All culture media were purchased from Huankai Microbial Sci. & Tech. Co. Ltd. (Guangzhou, China).

### 3.2. Extraction of Essential Oil

*PCR*s were subjected to hydro-distillation for 2 h, in a modified Clevenger-type apparatus, with a water-cooled oil receiver to reduce formation of artifacts due to overheating during hydro-distillation. The essential oils were collected over water, separated and dried over anhydrous sodium sulfate. They were stored at 4 °C prior to studies.

### 3.3. Gas Chromatography/Mass Spectrograph (GC/MS)

Desorption and analysis of volatile components were carried out on an Agilent 6890 GC system coupled with an Agilent MSD 5975 quadrupole mass spectrometer. The separation was achieved on two fused silica capillary columns: (1) DB-WAX (30 m × 25 mm i.d. × 25 μm film); (2) DB-5MS (30 m × 25 mm i.d. × 25 μm film). The carrier gas was helium with flow rate of 1.0 mL/min. A sample of 1.0 μL was injected, using split mode (split ratio, 1: 20). The injector temperature was set at 250 °C. The GC oven temperature was increased from 40 °C to 70 °C at a rate of 10 °C /min, then programmed at 3 °C/min to 190 °C, then at 10 °C/min to 250 °C. The temperature of mass spectrometer was 230 °C. The ionizing energy was 70 eV. All data were obtained by collecting the full-scan mass spectra within the scan range 40-500 amu.

### 3.4. Qualitative and Quantitative Analysis

Identification of compounds detected by GC/MS analysis was done by comparing mass spectra and retention indices (RI) with the authentic standards and published data, as well as by comparing their mass spectra with the National Institute of standards and Technology (NIST) MS spectral database [49]. RI was calculated using a mixture of *n*-alkanes as standards. Some volatile compounds’ regression equations were made and their concentrations were obtained from these equations. For the other volatile compounds, quantitative determinations were obtained by using cyclohexanone as an internal standard. Volatile compounds’ content was calculated from the GC-peak areas relating to the GC-peak area of the internal standard. Some results were expressed as follows: 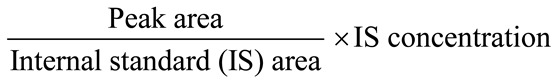


### 3.5. Scavenging of DPPH Radical

The effect of *PCR* on DPPH free radical was measured using the modified method of Shimada, Fujikawa, Yahara, and Nakamura [50]. A methanolic solution of DPPH (2.5 mL, 1 × 10^-4^ mol/L) was mixed with aliquots (0.5 mL) of different concentrations of sample in a tube. The reaction mixture was shaken well and incubated for 30 min at room temperature. Controls containing methanol instead of the antioxidant solution and blanks containing methanol instead of DPPH solution were also made. Vc was regarded as positive probe. The absorbance of the resulting solution was read at 517 nm against blank. The DPPH radical-scavenging activity was calculated as follows:

Scavenging activity (%) = [1 − (A_sample_ − A_blank_)]/A_control_ × 100

where A_sample_ is the absorbance of DPPH added to sample at 517 nm, A_blank_ is the absorbance of methanol added to sample at 517 nm, A_control_ is the absorbance of DPPH added to methanol (without sample) at 517 nm.

### 3.6. Ferric-Reducing Antioxidant Power (FRAP) Assay

For FRAP assay, the procedure followed the method of Benzie and Strain with some modifications [51]. The FRAP reagent included acetate buffer (pH 3.6), 10 mM TPTZ solution in 40 mM HCl, and 20 mM Trolox solution. The FRAP reagent was prepared and warmed to 37 °C in a water bath prior to use. FRAP reagent (900 μL) was mixed with H_2_O (90 μL) and test sample (30 μL), and the final dilution of the test sample in the reaction mixture was 1/34. The solution was used to perform the calibration curves. Results were expressed as μmol TE/g *PCR*.

### 3.7. ABTS·^+^ Assay

The antioxidant capacity of *PCR* was estimated according to the measurement described by Siddhuraju and Manian with some modifications [52]. ABTS was dissolved in water to a 7 mM concentration. ABTS radical cation (ABTS·^+^) was produced by the reaction of ABTS stock solution and 2.45 mM potassium persulfate (final concentration). Then the mixture was placed in the dark at room temperature for 12-16 h before use. Oxidation of the ABTS commenced immediately, but the absorbance was not maximal and stable until more than 6 h had elapsed. The radical was stable in this form for more than two days when stored in the dark at room temperature. Prior to assay, the solution was diluted in methanol, to an absorbance of 0.70 ± 0.02 at 734 nm and equilibrated at 30 °C. After addition of diluted ABTS·^+^ solution (1.0 mL) to test sample or Trolox standard (50 μL), the reaction mixture was incubated for 30 min at 30 °C. All the measurements were performed in triplicate and results were averaged. The unit was defined as the concentration of Trolox having the equivalent antioxidant activity expressed as μmol TE/g *PCR*.

### 3.8. Microbial Strains

The antimicriobial activity of the essential oil were individually tested against a panel of microorganisms, including Gram-positive bacteria: *Staphylococcus aureus* (*S. aureus*) (GIM 1. 142)*, Bacillus cereus* (*B. cereus*) (CMCC 63302)*, Bacillus subtilis* (*B. subtilis*) (ATCC 9372)*, Streptococcus faecalis* (*S. faecalis*) (ATCC 29212); Gram-negative bacteria: *Escherichia coli* (*E. coli*) (ATCC 25922), *Salmonella lignieres* (*S. lignieres*) (CMCC 50115), *Pseudomonas aeruginosa* (*P. aeruginosa*) (ATCC 9027), *Enterobacter cloacae* (*E. cloacae*) (CMCC 4350); fungi: *Aspergillus flavus* (*A. flavus*) (AS3. 3950), *Aspergullus niger* (*A. niger*) (ATCC 16404), *Debaryomyces. hansenii* (*D. hansenii*). All these microorganisms were purchased from Microbial Culture Collection Centre of Guangdong, China. Bacterial strains were cultured overnight at 37 °C in Nutrient agar (NA) while fungal strains were cultured overnight at 30 °C using Potato dextrose agar (PDA).

### 3.9. Disc Diffusion Assay

Determination of antimicrobial activity of essential oil of *PCR* was accomplished by agar disc diffusion method [53]. Briefly, suspension of tested microorganisms (100 µL), containing about 10^6^ colony-forming units (cfu)/mL of bacteria cells and 10^4^ cfu/mL spores of fungal strains spread on NA and PDA medium, respectively. The discs (6 mm in diameter) impregnated with the essential oil and placed on the inoculated agar. Disc soaked with amoxycillin (0.85 mg/mL) and flumequine (0.85 mg/mL) were served as a positive growth control for bacteria and fungi, respectively, while without samples were used as a negative control. The inoculated plates were incubated for 24 h at 37 °C for bacterial strains and 48 h at 30 °C for fungal strains. The diameters of inhibition zones were used as a measure of antimicrobial activity and each assay was conducted in triplicate.

### 3.10. Determinations of Minimum Inhibitory Concentration (MIC)

A broth microdilution method was used to determine the MIC [54]. Bacterial strains were cultured overnight at 37 °C in NB and the fungi were cultured overnight at 30 °C in SDB, and adjusted to a final density of 10^6^ cfu/mL. Dilutions series were prepared from 6 to 40 mg/mL of the essential oil in a 96-well microtitre plate, 160 µL of NB and SDB for bacteria and fungi, respectively, were added onto microplates and tested solution (20 µL). Then, 5 × 10^5^ cfu/mL of standard microorganism suspension (20 µL) were inoculated onto microplates. The inoculated plates were incubated for 24 h at 37 °C for bacterial strains and 48 h at 30 °C for fungal strains. The same test was performed simultaneously for the growth control (NB + DMSO) and sterility control (NB + DMSO + test oil). Amoxycillin was used as a reference compound for antibacterial and flumequine for antifungal activities. The growth was indicated by the presence of a white ‘‘pellet” on the well bottom.

#### 3.11. Statistical Analysis

All experiments were performed in triplicates and the experimental results represent treatment groups expressed as means ± SD. One-way analysis of variance (ANOVA) was used to compare the means, and the least significant difference (LSD) test showed the values statistically different at P < 0.05. Analysis was performed using SPSS 11.5 for windows.

## 4. Conclusions

*PCR* Chachi 2004 exhibited the highest yield rate of essential oil, while Chachi 2008 was the lowest. In the *PCRs*, 53 volatile compounds were identified, including terpenic hydrocarbons, alcohols, aldehydes, ketones and esters. D-limonene was the major constituent in *PCR*. The antioxidant capacity of *PCR* essential oil varied considerably with the duration of storage time and the essential oil possessed effective antimicrobial activity against Gram-positive bacteria (*S. aureus*, *B. subtilis*, *B. cereus*) in varying degrees, except *S. faecalis*, while had no effect on Gram-negative bacteria (*E. coli*, *E. cloacae*).

## Figures and Tables

**Table 1:** Yield rates and chemical composition of *PCR* essential oil at different storage times.

*No.*	*Compounds*	*KI*^ɑ^		*Concentration (g/L)*					ID
		DB-5MS	DB-WAX	Chachi 2008	Chachi 2004	Chachi 2001	Chachi 1998	Chachi 1994	
Yields rates of the essential oil (%)				0.96 ± 0.00	5.41 ± 0.09	4.29 ± 0.20	4.19 ± 0.05	2.13 ± 0.20	
1.	Acetone [23,24]^c^	–	811	0.013 ± 0.021	0.011 ± 0.001	0.012 ± 0.002	0.012 ± 0.001	0.008 ± 0.000	d,e,f
2.	3-Buten-2-ol, 2-methyl- [24]^c^	611	1039	0.281 ± 0.021	0.031 ± 0.005	0.350 ± 0.083	0.215 ± 0.004	0.195 ± 0.006	d,e,f
3.	Butanal, 3-methyl- [23,24,25]^c^	652	–	0.032 ± 0.008	0.008 ± 0.002	0.021 ± 0.022	0.007 ± 0.000	0.015 ± 0.002	d,e,f
4.	Butanal, 2-methyl- [24]^c^	662	–	0.010 ± 0.001	0.042 ± 0.006	0.003 ± 0.001	0.004 ± 0.000	0.003 ± 0.001	d,e,f
5.	2-Buten-1-ol, 3-methyl- [24,26]^c^	771	–	0.051 ± 0.014	0.084 ± 0.008	0.041 ± 0.009	0.018 ± 0.002	0.017 ± 0.001	d,e,f
6.	Furfural [24,25,26]^b^	828	–	0.711 ± 0.138	0.184 ± 0.023	0.117 ± 0.012	0.093 ± 0.002	0.085 ± 0.008	d,e,f
7.	2-Hexenal [25,27]^c^	849	–	0.014 ± 0.001	0.005 ± 0.001	0.010 ± 0.000	0.010± 0.001	0.012 ± 0.005	d,e,f
8.	Nonane^c^	900	–	0.014 ± 0.002	0.004 ± 0.001	0.011 ± 0.001	0.005 ± 0.001	0.006 ± 0.001	d,e
9.	Thujene [28]^c^	924	1023	1.669 ± 0.226	6.172 ± 0.828	6.310 ± 0.741	6.037 ± 0.590	5.870 ± 0.302	d,e,f
10.	α-Pinene [23,24,25]^b^	932	1019	2.825 ± 0.706	13.132 ± 1.702	15.053 ± 0.041	16.413 ± 1.176	17.945 ± 0.977	d,e,f
11.	Camphene [28]^c^	948	–	0.093 ± 0.011	0.188 ± 0.015	0.333 ± 0.009	0.338 ± 0.035	0.334 ± 0.055	d,e,f
12.	Furfural, 5-methyl- [29]^c^	956	–	0.031 ± 0.006	ND	0.0129 ± 0.002	0.021 ± 0.001	0.016 ± 0.004	d,e,f
13.	β-Phellandrene [28]^c^	971	1205	0.901 ± 0.138	1.322 ± 0.184	1.329 ± 0.232	0.678 ± 0.152	0.561 ± 0.033	d,e,f
14.	β-Pinene [25,28,29]^c^	977	1104	4.278 ± 0.367	11.317 ± 1.351	12.629 ± 0.078	12.953 ± 1.447	13.814 ± 0.902	d,e,f
15.	β-Myrcene [24,25,26,29]^b^	989	1157	61.112 ± 21.892	41.534 ± 10.124	42.647 ± 6.934	32.631 ± 3.893	20.443 ± 4.400	d,e,f
16.	α-Phellandrene [25,29]^c^	1007	–	1.066 ± 0.068	1.069 ± 0.148	1.118 ± 0.0625	1.061 ± 0.021	1.044 ± 0.040	d,e,f
17.	2-Carene [29]^c^	1017	–	0.938 ± 0.071	2.618 ± 0.289	1.983± 0.915	2.192 ± 0.123	2.013 ± 0.192	d,e,f
18.	Benzene, 1-methyl- 2-(1- methylethyl)- [28]^c^	1024	1266	1.343 ± 0.125	4.430 ± 0.719	9.730 ± 6.350	8.606 ± 1.459	10.246 ± 1.640	d,e,f
19.	D-Limonene [30]^b^	1033	1199	454.708 ± 32.664	393.136 ± 35.731	385.490 ± 63.149	386.604 ± 17.763	378.721 ± 44.626	d,e,f
20.	Ocimene [31]^c^	1049	1248	1.753 ± 0.315	1.535 ± 0.146	1.247 ± 0.372	1.380 ± 0.094	0.908 ± 0.020	d,e,f
21.	γ-Terpinene [24,28,29]^c^	1063	1242	35.164 ± 2.482	63.063 ± 5.316	64.038 ± 7.303	57.366 ± 6.664	50.083± 11.938	d,e,f
22.	Terpinolene [24,28]^b^	1086	1281	1.687 ± 0.271	11.933 ± 1.580	9.325 ± 1.977	9.159 ± 0.308	8.397 ± 1.386	d,e,f
23.	Benzene, 1-methyl- 4-(1- methylethenyl)-	1091	–	0.131 ± 0.026	0.606 ± 0.046	0.761 ± 0.210	0.672 ± 0.077	0.507 ± 0.107	d,e,f
	[26,28,30]^c^								
24.	Linalool [28,29]^c^	1100	1546	0.751 ± 0.073	1.712 ± 0.168	1.435 ± 0.108	0.662 ± 0.071	ND	d,e,f
25.	Nonanal [23,26,29]^c^	1105	–	0.103 ± 0.016	0.582 ± 0.029	0.532 ± 0.030	0.457 ± 0.006	0.447 ± 0.018	d,e,f
26.	β-Terpinol [32]^c^	1148	–	0.192 ± 0.022	0.469 ± 0.035	0.541 ± 0.153	0.531 ± 0.052	0.531 ± 0.006	d,e,f
27.	Citronellal [32]^c^	1151	–	0.116 ± 0.015	0.300 ± 0.043	0.288 ± 0.010	0.185 ± 0.032	ND	d,e,f
28.	L-4-Terpineol [33]^c^	1180	–	1.200 ± 0.221	1.614 ± 0.092	1.741 ± 0.031	1.984 ± 0.397	2.135 ± 0.001	d,e,f
29.	*p*-Menth-1-en-8-ol [26,29,32]^c^	1195	–	1.486 ± 0.078	1.982 ± 0.113	2.300 ± 0.078	2.698 ± 0.537	2.846 ± 0.079	d,e,f
*30.*	Decanal [27,29,32]*^b^*	1206	1492	1.391 ± 0.205	1.324 ± 0.136	1.181 ± 0.141	0.670 ± 0.093	0.603 ± 0.187	d,e,f
31.	Acetic acid, octyl ester [29]^c^	1210	–	0.125 ± 0.020	0.101 ± 0.010	ND	ND	ND	d,e,f
32.	*cis*-Carveol [29,32]^c^	1218	–	0.628 ± 0.132	0.391 ± 0.048	0.235 ± 0.005	0.679 ± 0.019	0.698 ± 0.063	d,e,f
33.	*trans*-Carveol [29,32]^c^	1232	–	0.065 ± 0.003	0.149 ± 0.006	0.259 ± 0072	0.318 ± 0.012	0.339 ± 0.044	d,e,f
34.	Carvone [32]^c^	1244	–	0.101 ± 0.025	0.273 ± 0.009	0.214 ± 0.020	0.311 ± 0.006	0.337 ± 0.067	d,e,f
35.	Geraniol [26,29,32]^c^	1250	–	0.131 ± 0.012	ND	ND	ND	ND	d,e,f
36.	Perillaldehyde [32]^c^	1276	1766	0.071 ± 0.011	0.426 ± 0.020	0.374 ± 0.072	0.074 ± 0.014	0.097 ± 0.026	d,e,f
37.	Carvacrol [29]^c^	1292	–	0.246 ± 0.034	0.677 ± 0.058	ND	ND	ND	d,e,f
38.	Vinylguaiacol [34]^c^	1310	–	0.951 ± 0.069	0.254 ± 0.025	0.330 ± 0.073	0.308 ± 0.042	0.306 ± 0.018	d,e,f
39.	δ-Elemene [35]^c^	1336	–	1.424 ± 0.135	ND	ND	ND	ND	d,e,f
40.	α-Cubebene [36]^c^	1347	–	0.115 ± 0.020	0.196 ± 0.014	0.030 ± 0.001	0.030 ± 0.005	0.035 ± 0.004	d,e,f
41.	Nerol acetate [29,32]^c^	1359	–	1.307 ± 0.177	0.050 ± 0.006	0.0630 ± 0.011	0.098 ± 0.016	0.110 ± 0.009	d,e,f
42.	β-Elemene [29,35]^c^	1388	–	2.880 ± 0.172	0.044 ± 0.002	ND	0. 087 ± 0.018	0.109 ± 0.020	d,e,f
43.	β-Caryophyllene [28,29,32]^b^	1416	–	0.725 ± 0.071	0.710 ± 0.094	0.672 ± 0.207	0.933 ± 0.190	1.092 ± 0.036	d,e,f
44.	α-Caryophyllene [28,29]^c^	1452	–	0.981 ± 0.089	0.087 ± 0.008	0.091 ± 0.007	0.112 ± 0.026	0.136 ± 0.018	d,e,f
45.	Germacrene-D [28,29,36]^c^	1478	–	3.265 ± 0.203	0.096 ± 0.000	0.085 ± 0.015	0.062 ± 0.025	0.055 ± 0.015	d,e,f
46.	Valencene [28,29,32]^c^	1489	–	0.735 ± 0.049	0.031 ± 0.005	0.032 ± 0.003	0.040 ± 0.003	0.039 ± 0.001	d,e,f
47.	Bicyclogermacrene [28]^c^	1492	–	0.798 ± 0.063	0.281 ± 0.037	0.270 ± 0.067	0.269 ± 0.046	0.310 ± 0.035	d,e,f
48.	α-Muurolene [24]^c^	1495	–	0.258 ± 0.032	0.023 ± 0.002	0.032 ± 0.008	0.033 ± 0.003	0.048 ± 0.020	d,e,f
49.	α-Bulnesene [29]^c^	1498	–	0.334 ± 0.035	ND	ND	ND	ND	d,e,f
50.	α-Farnesene [37]^c^	1503	1741	6.116 ± 0.381	2.021 ± 0.199	2.013 ± 0.317	1.439 ± 0.167	1.287 ± 0.256	d,e,f
51.	δ-Cadinene [29,36,37]^c^	1515	–	1.841 ± 0.241	0.227 ± 0.008	0.246 ± 0.023	0.322 ± 0.057	0.422 ± 0.114	d,e,f
52.	Cadinadiene-1,4 [38]^c^	1529	–	0.038 ± 0.003	ND	ND	ND	ND	d,e,f
53.	Elemol [37]^c^	1545	–	0.139 ± 0.017	0.020 ± 0.001	0.019 ± 0.001	0.053 ± 0.012	0.066 ± 0.011	d,e,f

ND: not detected.

^a^ KI: Kovats indices obtained using series of n-alkanes on DB-5MS and DB-WAX column.

^b^ Concentration are obtained from these regression equations on DB-5MS column.

^c^ Concentration are obtained on DB-5MS column, (Peak area/IS area) × IS concentration (*n* = 3).

ID: identification by ^d^ mass spectrum, ^e^ KI and ^f^ references.

**Table 2:** The concentration range, regression equations, R^2^, recovery for the standard compounds.

*Compounds*	*Retention time*	*Concentration* *range (g/L)*	*Regression* *equation*	*R^2^*	Recovery range (%)
Furfural	5.409	0.023-2.320	*Y* = 1E + 07*X* + 672948	0.9983	89.3
α-Pinene	7.827	1.718-17.182	*Y* = 2E + 07*X* + 2E + 08	0.9588	104.2
β-Myrcene	9.443	15.820-791.000	*Y* = 3E + 06*X* + 4E + 08	0.9947	88.5
D-Limonene	11.050	84.020-840.200	*Y* = 9E + 06*X* + 2E + 09	0.9975	90.3
Terpinolene	12.775	0.861-86.100	*Y* = 1E + 07*X* + 8E + 07	0.9769	84.9
Decanal	17.640	0.415-8.300	*Y* = 2E + 07*X* − 701044	0.9976	113.7
β-Caryophyllene	26.557	0.450-8.995	*Y* = 2E + 07*X* + 7E + 06	0.9977	106.4

*Y*: the volatile compound peak area; *X*: the volatile compound concentration.

**Table 3:** The antioxidant activities of *PCR* essential oil at different storage time.

*Antioxidant activities*	*Chachi 2008*	*Chachi 2004*	*Chachi 2001*	*Chachi 1998*	*Chachi 1994*
DPPH IC_50_ (mg/mL)	6.30 ± 0.50a	13.33 ± 0.56c	8.45 ± 0.51b	9.24 ± 0.59b	13.40 ± 0.46c
FRAP (μmol TE/g *PCR*)	11.36 ± 1.50a	15.79 ± 1.62b	17.50 ± 1.48b	21.61 ± 3.80c	26.89 ± 1.11d
ABTS (μmol TE/g *PCR*)	21.23 ± 1.09b	21.59 ± 1.08b	26.48 ± 1.36d	24.22 ± 0.74c	16.71 ± 1.40a

Values are means ± standard deviation of three separate experiments. Different letters (a, b, c) in a row indicate significant differences (p < 0.05).

**Table 4:** The antimicrobial activity of *PCR* essential oil at different storage time.

*Tested organism*	*Essential oils*					*Amoxicillin*	Flumequine
	Chachi 2008	Chachi 2004	Chachi 2001	Chachi 1998	Chachi 1994		
Diameter of inhibition zone (mm)^A^							
*S. lignieres*	7.8 ± 0.4a	8.4 ± 0.8a	9.3 ± 0.8a	8.7 ± 0.7a	8.2 ± 0.8a	18.0 ± 1.5b	
*E. coli*	6.0 ± 0.0a	6.0 ± 0.0a	6.0 ± 0.0a	6.0 ± 0.0a	6.0 ± 0.0a	11.4 ± 0.3b	
*S. aureus*	8.9 ± 1.3a	11.3 ± 1.4a	16.9 ± 2.7b	9.4 ± 1.0a	10.4 ± 0.7a	44.8 ± 3.0c	
*B. subtilis*	9.8 ± 1.6a	13.7 ± 2.8ab	22.7 ± 2.4c	12.2 ± 1.8a	17.1 ± 3.9b	56.2 ± 1.9d	
*P. aeruginosa*	7.8 ± 0.4a	7.4 ± 0.2a	7.7 ± 0.3a	8.6 ± 1.0ab	7.4 ± 0.2a	9.3 ± 0.6b	
*B. cereus*	9.3 ± 1.2a	8.4 ± 0.5a	8.7 ± 1.2a	8.1 ± 0.6a	9.0 ± 0.9a	15.3 ± 1.2b	
*E. cloacae*	6.0 ± 0.0a	6.0 ± 0.0a	6.0 ± 0.0a	6.0 ± 0.0a	6.0 ± 0.0a	6.0 ± 0.0a	
*S. faecalis*	6.0 ± 0.0a	6.0 ± 0.0a	6.0 ± 0.0a	6.0 ± 0.0a	6.0 ± 0.0a	33.1 ± 1.7b	
*A. flavus*	13.6 ± 0.8a	13.9 ± 1.3a	14.1 ± 0.9a	14.6 ± 1.2a	11.4 ± 2.6a		18.1 ± 2.9b
*A. niger*	6.0 ± 0.0a	6.0 ± 0.0a	6.0 ± 0.0a	6.0 ± 0.0a	6.0 ± 0.0a		6.0 ± 0.0a
*D. hansenii*	10.5 ± 0.5a	10.8 ± 0.9a	10.9 ± 1.2a	10.7 ± 0.8a	10.4 ± 0.6a		10.6 ± 0.4a
Minimum inhibitory concentration (mg/mL)							
*S. lignieres*	0.12 ± 0.02b	0.12 ±0.02b	0.12 ± 0.02b	0.12 ± 0.02b	0.1 ± 0.01b	0.03 ± 0.0a	
*E. coli*	NT	NT	NT	NT	NT	NT	
*S. aureus*	0.12 ± 0.02b	0.12 ± 0.02b	0.12 ± 0.02b	0.12 ± 0.02b	0.12 ± 0.02b	0.0±0.0a	
*B. subtilis*	0.03 ± 0.00b	0.03 ± 0.0b	0.03 ± 0.0b	0.03 ± 0.0b	0.03 ± 0.0b	0.0 ± 0.0a	
*P. aeruginosa*	NT	NT	NT	NT	NT	NT	
*B. cereus*	0.06 ± 0.00a	0.06 ± 0.00a	0.06 ± 0.0a	0.06 ±0.0a	0.06 ± 0.0a	0.05 ± 0.0a	
*E. cloacae*	NT	NT	NT	NT	NT	NT	
*S. faecalis*	NT	NT	NT	NT	NT	0.02 ±0.0	
*A. flavus*	0.06 ± 0.00b	0.06 ±0.0b	0.06 ± 0.0b	0.06 ± 0.0b	0.06 ± 0.0b		0.0 ± 0.0a
*A. niger*	NT	NT	NT	NT	NT		0.02 ± 0.0
*D. hansenii*	0.03 ± 0.00a	0.03 ± 0.0a	0.03 ± 0.0a	0.03 ± 0.0a	0.03 ± 0.0a		0.02 ± 0.0a

NT: not tested. Values are means ± standard deviation of three separate experiments. Different letters in a row indicate significant differences. A diameter of inhibition zone (mm) including disc diameter of 6 mm.
